# Efficient Recovery of Vanadium and Titanium from Domestic Titanomagnetite Concentrate Using Molten Salt Roasting and Water Leaching

**DOI:** 10.3390/ma16216918

**Published:** 2023-10-27

**Authors:** Ha Bich Trinh, Seunghyun Kim, Jaeryeong Lee, Seokhoon Oh

**Affiliations:** Department of Integrated Energy and Infra System, Kangwon National University, Chuncheon 24341, Republic of Korea; hab.trinh@gmail.com (H.B.T.); rlatmdgus930@kangwon.ac.kr (S.K.)

**Keywords:** vanadium, titanium, titanomagnetite concentrate, molten roasting, water leaching

## Abstract

The traditional roasting technique using sodium salts in vanadium production has been disadvantageous due to the large consumption of energy and the emission of harmful gases. A modified process using molten salt roasting and water leaching to extract vanadium and titanium from domestic titanomagnetite concentrate was investigated. The roasting process was performed under optimal conditions: the weight ratio between the sample and NaOH of 1:1, the temperature of 400 °C, and the experiment time 90 min, and the conversion of vanadium could be maximized to 90%. The optimization of water leaching (at 60 °C for 90 min with a pulp density of 0.05 g/mL) could extract 98% of the vanadium from the roasted products into the solution, leaving titanium and iron remaining in the residue. Further purification of vanadium and titanium using the precipitation/hydrolysis process followed by calcination obtained the final products V_2_O_5_ and TiO_2_ with high purities of 90% and 96%, respectively. A potential approach with modification of the roasting stage using NaOH was proposed, which was not only efficient to selectively extract the value metals from the titanomagnetite but also eco-friendly based on the reduction in energy consumption and emission of harmful gases.

## 1. Introduction

Vanadium and its compounds have been used widely in a variety of important fields, mainly in: steelmaking, petrochemical industry, non-ferrous alloys, chemical production, and batteries [1,2,3,4,5,6]. Recently, the increasing global consumption of vanadium is not only derived from the necessity of strength improvement in stainless steel and vanadium-titanium alloys but also from the rise in the use of vanadium in lithium batteries for energy storage at a large scale [7,8,9]. Therefore, consideration should be given to maintain adequate supplies of vanadium and develop a more sustainable production. Generally, vanadium exists in the form of oxides, sulfides, or phosphates associated with other metals (iron, titanium, lead, aluminum, zinc, chromium…) in natural ores such as titanomagnetite, patronite, vanadinite, descloizite, and carnotite [10]. Although titanomagnetite has been the major resource for vanadium metallurgical processes, the initial contents of vanadium are critically low (less than 1%) to supply for direct production [11]. It is necessary to enrich the content of vanadium using physical separations or to exploit the slags bearing vanadium generated during the smelting of titanomagnetite in the iron/steelmaking process by using a blast furnace or the direct reduction technique [4,5,6]. The vanadium contents in the collected slags can be enhanced by 5–20% after the smelting and converting stage depending on the feed resources, process efficiency, and the utilized techniques [4,5]. However, the mineralogical compositions of vanadium are normally present in the stable spinel phases (Fe, Mn)_2_(V, Ti)O_4_ or (Fe, Mg, Mn)(V, Cr)_2_O_4_, which leads to the inefficiency of direct leaching from titanomagnetite concentrates or vanadium-bearing slags without the assistance of pressure, microwave, or ultrasound [5]. The conventional approach is the conversion of vanadium in the spinel phases to more leachable phases by using pre-roasting with or without additives for further dissolution in water, acidic or alkaline media.

The traditional roasting technique involving the presence of sodium salts at high temperatures (NaCl 750–850 °C, Na_2_CO_3_ 800–1000 °C or Na_2_SO_4_ 1200–1250 °C) has been assigned as the most popular process with the longest history of development in vanadium production [5]. However, the emission of harmful gases (CO_2_, SO_2_ or Cl_2_) and the large consumption of energy are the disadvantageous problems of sodium salt roasting, which leads to the investigation of alternative roasting techniques such as molten salt roasting, calcia roasting, magnesia roasting, or microwave-assisted roasting. Molten roasting using NaOH with a mass ratio of 1:1, at 500 °C for 60 min followed by water leaching at 50 °C, for 20 min with a pulp density of 20% could effectively leach out 96.6% of vanadium from titanomagnetite concentrates [12]. Furthermore, modification of the NaOH roasting technique by using a concentrated solution of NaOH, KOH, or a combination of NaOH and NaNO_3_, namely liquid oxidation technology, was demonstrated to obtain an excellent extraction efficiency of 95% while the operated temperature was significantly reduced to 200–400 °C [13,14]. Binary sodium salts (NaOH-NaNO_3_) roasting with microwave assistance not only performed efficient recovery of vanadium 94.1%, but also reduced the roasting duration since the microwave power could improve the heating speed and accelerate the oxidation reactions [15]. Calcification roasting (at 850 °C for 120 min) followed by sulfuric acid leaching was found to reach the maximum vanadium leachability of 93% [16]. MgO-based roasting was employed to effectively extract ~95% of vanadium under optimal conditions: a temperature of 900 °C, a duration of 90 min, and a Mg/V ratio of 0.6 [17]. Recently, direct reduction roasting with carbon sources such as graphite powder, calcium carbonate, or a mixture of (H_2_ + CO) has been investigated to develop a cleaner and more productive process for vanadium extraction [18,19,20,21]. Although there are a variety of alternative approaches to modify roasting techniques for vanadium production, molten salt roasting with the presence of sodium hydroxide or binary salts has been evaluated as the most promising technique. It is evidently more advantageous than other roasting techniques with regard to significantly reducing the impact on the environment based on the exclusion of harmful gases while maintaining the sufficient efficiency of vanadium recovery. Moreover, the avoidance of using carbon-sources and lower roasting temperatures can reduce the release of CO_2_ gas and the high consumption of energy, which has been a long-term concern due to global warming. Therefore, it is worth developing such a “greener process” to produce vanadium and other valuable metals to solve the issue of balance and direct efforts to more sustainable metallurgy.

In the present work, roasting using sodium hydroxide followed by the water leaching of domestic titanomagnetite concentrate was investigated to maximize the recovery efficiency of vanadium. The vanadium in leaching liquor was precipitated as ammonium polyvanadate by the addition of ammonium chloride, and further calcined to generate the product V_2_O_5_. Meanwhile, the collected residue after water leaching was consequently dissolved in sulfuric acid H_2_SO_4_ solution and then hydrolyzed to form the precipitation of H_2_TiO_3_ and converted to the product TiO_2_ using calcination. The modification of the roasting technique by using NaOH as the substitute for sodium salts and the avoidance of using carbon sources in the roasting stage is an alternative approach to reduce the harmfulness to the environment, the consumption of energy, and the emission of CO_2_ while still being an effective process to selectively recover value materials (V and Ti) with high purity from titanomagnetite concentrate.

## 2. Materials and Methods

### 2.1. Materials

The titanomagnetite concentrate in this study was supplied by SAMYANG M. B. T from Gwan-in Mine, Gyeonggi-do, Republic of Korea. The wet digestion method was used to analyze the chemical compositions of the concentrate (listed in Table 1). Sodium hydroxide (NaOH, purity 97%, Junsei Chemical Co., Ltd., Tokyo, Japan) was used as the roasting additive, while ammonium chloride (NH_4_Cl, purity 99%, Oriental Chemical Industries Co., Ltd., Seoul, Republic of Korea) was employed to precipitate vanadium in the solution after water leaching with pH adjustment by hydrochloric acid (HCl, 35%, Junsei Chemical Co., Ltd., Tokyo, Japan). Sulfuric acid (H_2_SO_4_, 95%, Junsei Chemical Co., Ltd., Tokyo, Japan) was used to dissolve the titanium-bearing residue before hydrolysis to purify titanium.

### 2.2. Methods

Ten grams of titanomagnetite concentrate was mixed with NaOH powered at a ratio varied from 0.5:1 to 2:1, and then roasted in an electric furnace (KF-S-1001-1000, Korea Furnace Development Co., Seoul, Republic of Korea) to select the optimal conditions at the temperature range of 100–400 °C and the duration of 10–120 min. Water leaching experiments were performed in a glass beaker at a fixed stirring speed of 250 rpm using a magnetic stirrer bar. A specific amount of roasted product was dissolved in a certain volume of water, maintained at the desired conditions of the investigated experiment, and filtrated to obtain the leach liquor for the analysis of metal contents. The vanadium in the solution was precipitated using NH_4_Cl with pH adjustment by HCl solution. The leach residues were properly washed and further dissolved in H_2_SO_4_ solution for the hydrolysis of titanium. The sample, the roasted products, and the leach residues were collected to be analyzed by X-ray diffraction (XRD, D2 Phaser, Bruker, Republic of Korea).

The metal content was analyzed with an inductively coupled plasma atomic-emission spectrometer (AES-ICP, OPTIMA 7300DV, Perkin Elmer, Seoul, Republic of Korea). The ICP results were used to estimate the metal leaching efficiency as:

(1) $$\%\;\mathrm{Leaching}=\left(\frac{M_{L}}{M_{S}}\right)\;\times \;100$$

where M_S_ and M_L_ are the metal masses in the initial feed sample and the leach liquor, respectively.

## 3. Results

### 3.1. NaOH Roasting to Decompose the Vanadium Spinel in the Titanomagnetite Concentrate

#### 3.1.1. Effect of Roasting Temperature and Time

The influence of temperature and time on conversion efficiency during NaOH roasting was investigated by varying in the range of 100–400 °C from 10 to 120 min with the fixed weight ratio of 1:1 between the NaOH and the sample. Subsequently, water leaching of the roasted products was carried out at 60 °C for 120 min and a pulp density (PD) 0.01 g/mL to evaluate the conversion of vanadium by NaOH roasting based on the obtained leachability.

**Figure 1:**
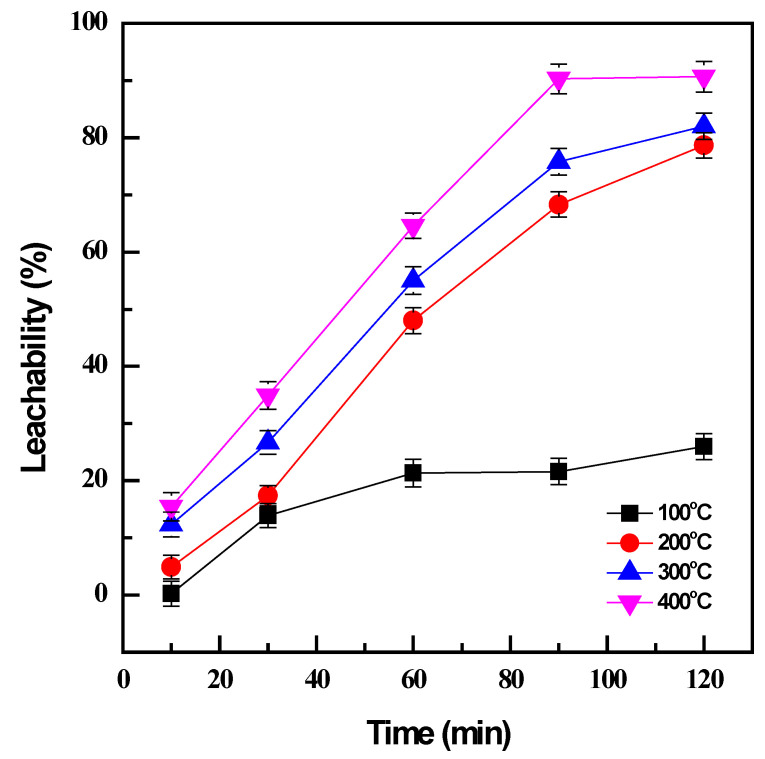
Effect of roasting temperature and time on vanadium conversion (roasting conditions: 100–400 °C, 10–120 min, and NaOH: sample ratio 1:1; water leaching conditions: 60 °C, 120 min, and PD 0.01 g/mL).

At low temperatures, roasting was not effective to transform the sample to the desired products; only 21% of vanadium was converted at 100 °C after 60 min (Figure 1). A higher temperature is evidently essential since an increase in roasting temperature from 100 °C to 400 °C could significantly enhance the conversion efficiency from 21% to 64% for the same roasting time. The increase in roasting temperature not only accelerated the oxidation reactions but also supported the mass transfer by reducing the viscosity of NaOH molten phases [14]. Although the decomposition of vanadium spinel in the titanomagnetite concentrate was more favorable at higher temperatures, reasonable conditions should be selected to avoid the large consumption of energy while still maintaining productive efficiency. Therefore, the roasting time was considered as well as the roasting temperature. The prolonged roasting duration did not improve the roasting effectiveness at low temperatures; the conversion efficiency was less than 30% after 120 min roasting at 100 °C. However, there was a considerable enhancement from 12% to 68% at a higher roasting temperature of 200 °C when the roasting time increased from 10 min to 90 min, and 90% roasting efficiency could be obtained at 400 °C with the same duration. Further improvement was not observed after the roasting time was extended to 120 min. Consequently, the optimal conditions were managed at 400 °C and 90 min to achieve the effective conversion of vanadium by NaOH roasting.

#### 3.1.2. Effect of NaOH Dosage

The conversion of vanadium by roasting required a certain high dosage of NaOH [6,14]; therefore, the amount of NaOH as the additive was optimized by mixing with the titanomagnetite concentrate at the mass ratio (NaOH: sample) varied from 0.5:1 to 2:1.

**Figure 2:**
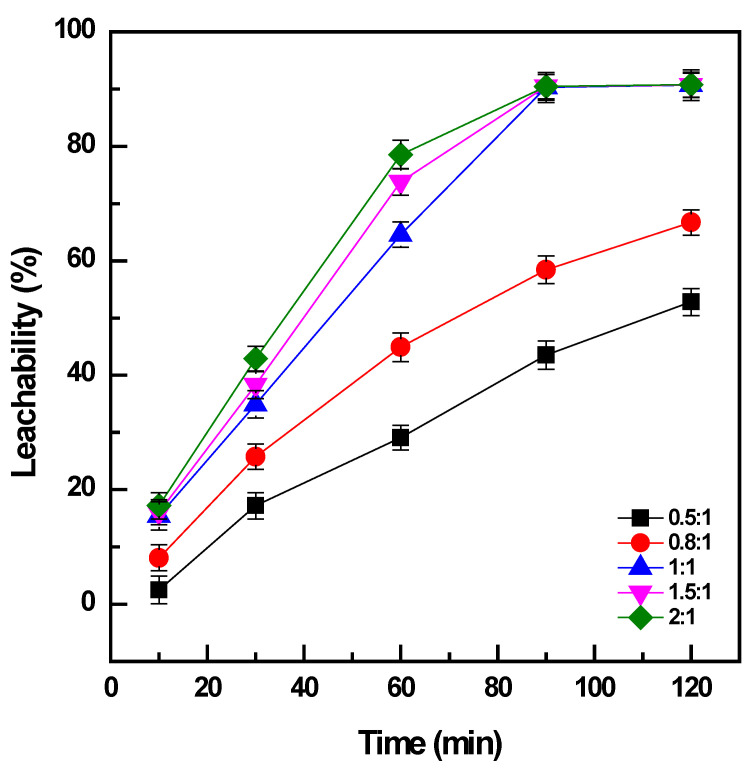
Effect of NaOH dosage on vanadium conversion (roasting conditions: 400 °C, 10–120 min, and NaOH: sample ratio from 0.5:1 to 2:1; water leaching conditions: 60 °C, 120 min, and PD 0.01 g/mL).

The addition of NaOH to the sample at a ratio of 0.5:1 could convert a minor amount of the vanadium in the concentrate: ~30% for 60 min (Figure 2). Under the same conditions, the increase in NaOH amount to the ratio of 1:1 promoted the conversion of vanadium to 65%, and it reached a maximum efficiency of 90% after 90 min. The addition of NaOH at a ratio of more than 1:1 was effective for a short duration of roasting less than 60 min; however, the enhancement was not significant after roasting for 90 min. When the addition of NaOH was not adequate, it could generate sodium vanadate with an Na:V ratio of less than 1 as the roasted product, which had lower solubility than the other compounds with a high Na:V ratio forming at high doses of NaOH, such as sodium pyrovanadate or orthovanadate [4]. Moreover, the increase in NaOH addition could decrease the viscosity of the molten phase and support the mass transfer of oxygen gas and the sample particles in the media during roasting [14]. Therefore, the amount of NaOH should be sufficiently maintained to form the soluble products of vanadates and accelerate the roasting reactions in the molten phase. Consequently, the weight ratio between NaOH and the sample of 1:1 was selected as the optimal dosage for further investigations. The dissolution of titanium and iron was not observable, which was attributed to the alkaline media of the solution during water leaching (with the pH in the range of 12.5 to 12.6).

### 3.2. Water Leaching of Roasted Products

#### 3.2.1. Effect of Leaching Temperature and Time

**Figure 3:**
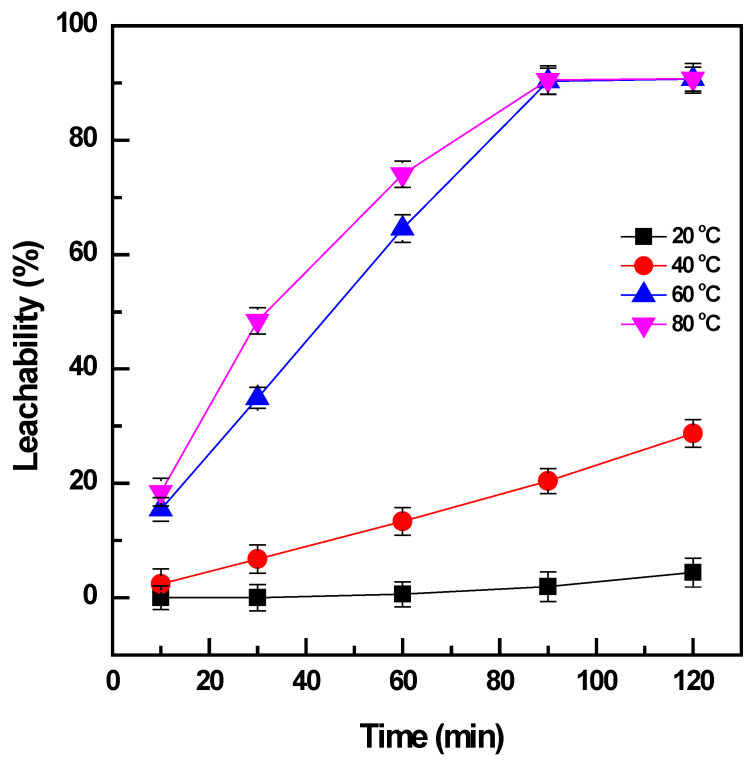
Effect of temperature and time on vanadium leachability (roasting conditions: 400 °C, 90 min, and NaOH: sample ratio 1:1; water leaching conditions: 20–80 °C, 10–120 min, and PD 0.01 mg/L).

The effect of temperature on the leaching of vanadium from the roasted products was studied in a range of 20 to 80 °C when the pulp density was maintained at 0.01 mg/L and the time was varied from 10 to 120 min. The increase in temperature had a significant influence on the leachability of the vanadium (Figure 3). The roasted products were not effectively dissolved at the low temperature of 20 °C, and vanadium was slightly extracted (~4%) after leaching for 120 min. The higher leaching temperature of 40 °C could enhance the dissolution of vanadium from 4% to 29% for the same duration of leaching; however, it was not sufficient to obtain the total leaching of vanadium. The temperature was continuously increased to 60 °C, and there was a considerable improvement in vanadium extraction to ~91%. Further enhancement of vanadium leachability was not observed when the temperature reached 80 °C and the leaching time increased to more than 90 min. Therefore, the temperature of 60 °C was selected to obtain the effective extraction of vanadium from the roasted products.

The leaching of vanadium from the roasted products was assumed to follow several kinetic equations, as listed in Table 2 [22]. The leachability with variation in temperature and time was used to evaluate the most suitable kinetic model. The fitting results presented a good linear relationship regarding the kinetic equations, with the average value of the regression coefficient R^2^ being more than 0.9 in all cases of the kinetic models. However, the significant influence of temperature on the dissolution of vanadium and the high value of the activation energy *E_a(leaching)_* > 40 kJ/mol indicated that the leaching of vanadium followed the chemical-controlled model [23]. Therefore, the equation 1 − (1 − *x*)^1/3^ = *k*_c_ × t was selected to interpret the vanadium leaching mechanism. The chemical control model with a high value of activation energy explained the dependence of vanadium leaching on temperature, which is expressed by the significant enhancement of efficiency with the increase in temperature.

#### 3.2.2. Effect of Pulp Density

The ratio between the roasted products and the volume of the leaching solution can have a certain influence on the leaching of vanadium; hence, it was investigated by varying the pulp density (PD, solid/liquid ratio of roasted products and water volume, g/mL) from 0.01 to 0.1 while the leaching time was varied in a range of 10 to 120 min and the leaching temperature was fixed at 60 °C.

**Figure 4:**
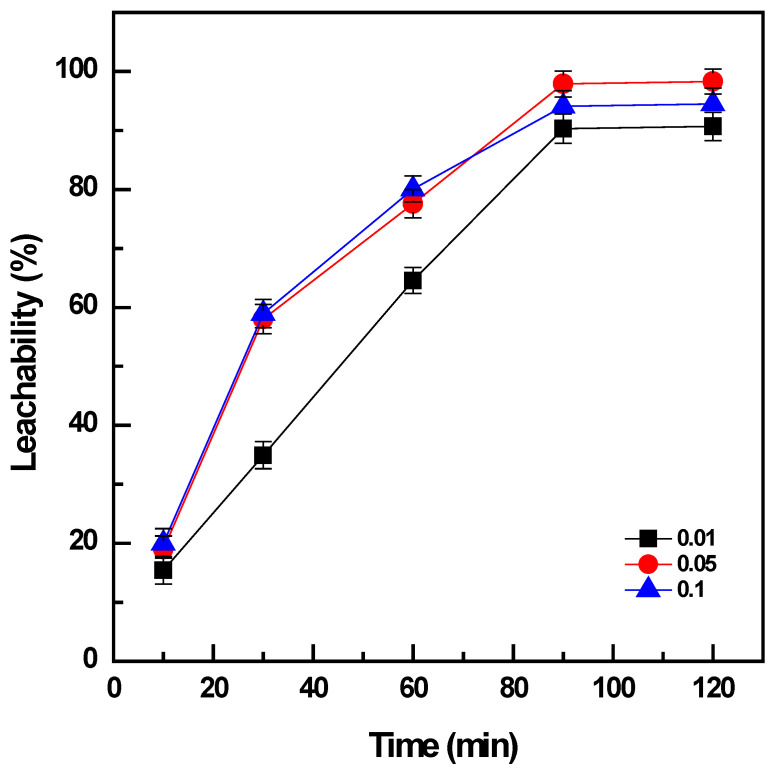
Effect of pulp density on vanadium leachability (roasting conditions: 400 °C, 90 min, and NaOH: sample ratio 1:1; water leaching conditions: 60 °C, 10–120 min, and PD 0.01 to 0.1 g/mL).

The results indicated that the increase in PD from 0.01 to 0.1 promoted the leachability of vanadium from 91% to ~98% after leaching for 90 min (Figure 4). However, the double amount of the solid phase, PD = 0.1 g/mL, produced a slight decrease on the vanadium dissolution to 94% for the same duration of leaching. The increase in the duration of leaching from 10 to 90 min could enhance the extraction efficiency of vanadium under the condition of PD = 0.05 g/mL from 20% to ~98%, and the same tendency was observed for PD = 0.01 or 0.1 mg/L. There was no further improvement in the leachability of vanadium when the leaching time was extended to more than 90 min. Consequently, water leaching was optimized under the conditions: 60 °C, PD = 0.05 g/mL, and 90 min to obtain the maximum extraction of 98% vanadium from the roasted products.

### 3.3. Preparation and Characterization of the Final Products (V_2_O_5_ and TiO_2_)

NaOH roasting and water leaching under optimal conditions could extract 98% of the vanadium into the solution (final pH = 12.5). In contrast, the dissolution of titanium and iron were critically low and remained in the residue. Purification of vanadium was necessary to separate it from other impurities, and precipitation using ammonium salts was the suitable method for the solution obtained from NaOH roasting and water leaching [5]. Although vanadium could be directly precipitated in alkaline media, the acidic media was preferred due to the higher purity of the final product and the less significant effect of Na on the precipitation efficiency of vanadium [24]. Therefore, the solution was initially adjusted from pH = 12.5 to acidic media pH = 2 using HCl solution, and vanadium was precipitated under the following conditions: NH_4_Cl as the precipitant, a temperature of 90 °C, and a duration of 120 min.

**Figure 5:**
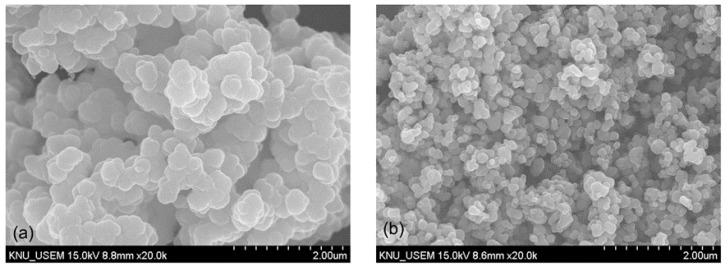
SEM images of precipitated products; (**a**) morphology of the vanadium precipitate using NH_4_Cl and (**b**) morphology of the titanium precipitate using hydrolysis.

The precipitation of vanadium was characterized using a scanning electron microscope (SEM, Hitachi S–4800, Tokyo, Japan), which showed the spherical structure with irregular edges of the precipitated product (Figure 5a). The solid product was collected and properly washed with NH_4_Cl solution before calcination at 550 °C for 120 min to produce the vanadium pentoxide V_2_O_5_. The residue after water leaching was further dissolved in H_2_SO_4_ solution of 2.0 M to selectively extract titanium from iron by using the hydrolysis process (1 g seed TiO_2_, 100 °C, 120 min) [25]. The SEM image presented the relatively uniform spherical shape and radius of the product particles (Figure 5b). The H_2_TiO_3_ precipitate was filtrated, washed, and calcinated at 800 °C to produce the titanium dioxide TiO_2_. The chemical composition analysis demonstrated that the purity of V_2_O_5_ was relatively low at 90%, and the other impurities were Al_2_O_3_ 5.7% and SiO_2_ 3.5%. However, TiO_2_ could be obtained with 96% purity and a low level of other impurities (Fe_2_O_3_ 3.8%).

### 3.4. Phase Transformation during the Recovery Process

**Figure 6:**
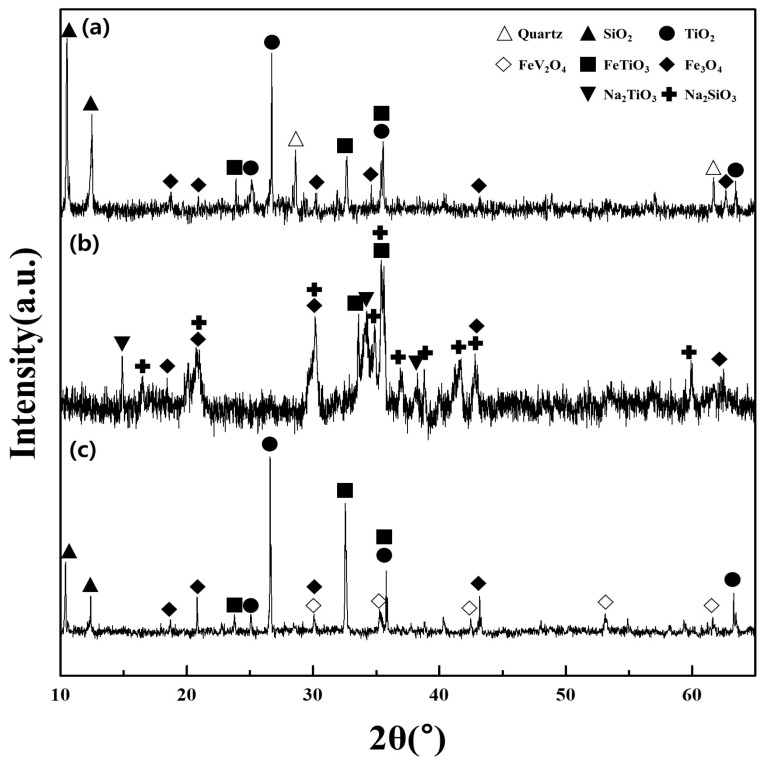
XRD patterns of samples and products during the recovery process; (**a**) titanomagnetite concentrate sample; (**b**) roasted products with NaOH; and (**c**) titanium-bearing residue after water leaching.

The major components of the titanomagnetite concentrate included Ilmenite FeTiO_3_, Fe_3_O_4_, TiO_2_, and SiO_2_ (Figure 6a). The vanadium was not detected in the XRD pattern of the concentrate sample due to the minor content present (4.18%). However, the peaks of the spinel phase FeV_2_O_4_ were observed in the patterns of the residue after water leaching. This provided indirect evidence for the phase of V in the initial concentrate since the spinel phase FeV_2_O_4_ was not completely converted and therefore appeared in the residue. After roasting using NaOH under optimal conditions, the vanadium in the spinel phase FeV_2_O_4_ could be converted into several vanadate products (NaVO_3_ or Na_4_V_2_O_7_) since the amount of additive NaOH exceeded the amount of vanadium in the sample (Equations (2) and (3) in Table 3) [4]. The other roasted products were Na_2_SiO_3_ and Na_2_TiO_3_ produced by reactions between NaOH and TiO_2_, SiO_2_, while the phases of Fe_3_O_4_ and FeTiO_3_ were maintained in the residue (Figure 6b and Equations (4) and (5)). In the water leaching stage, the vanadate compounds were dissolved to form a stable phase of HVO_4_^2−^ with regard to the redox potential 377 mV and the pH 12.5 of the leaching solution [4] (Equations (6) and (7)). The titanium and iron were observed as the phases of FeTiO_3_, Fe_3_O_4_, and TiO_2_ in the residue (Figure 6c and Equation (8)). Generally, the vanadium in the solution after the water leaching was transferred to the polymer ions such as V_10_O_28_^6−^, HV_10_O_28_^5−^, H_2_V_10_O_28_^4−^ in the acidic media and precipitated using NH_4_Cl [6]. In the present study, the redox potential (1105 mV) and the pH = 2 of the solution after the pH adjustment indicated that the vanadium existed as H_2_V_10_O_28_^4−^ and could be converted to (NH_4_)_2_V_6_O_16_ (Equation (9)). The titanium-bearing residue was dissolved into TiOSO_4_ using H_2_SO_4_ solution of 2.0 M (redox potential 980 mV and pH < 0), and titanium was separated from the solution by hydrolysis as H_2_TiO_3_ (Equations (10)–(12)). Finally, the vanadium pentoxide and titanium dioxide were prepared by calcination of the solid products obtained from the precipitation stage (Equations (13) and (14)).

### 3.5. Comparison between Modified and Conventional Roasting Processes

**Figure 7:**
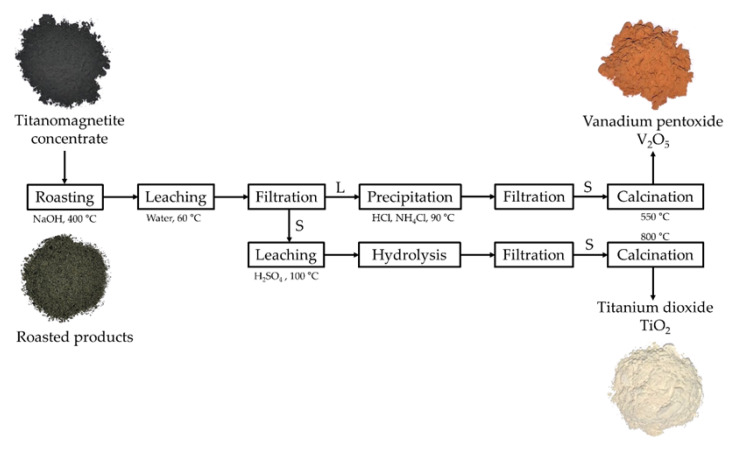
Flowsheet of the NaOH roasting and water leaching process to recover vanadium and titanium from the titanomagnetite concentrate (S: solid phase; L: liquid phase in the filtration stage).

An efficient process to recover the value metals vanadium and titanium from domestic titanomagnetite is presented (Figure 7). The conventional roasting technique using sodium salts as additives required high-temperature NaCl at 750–850 °C, Na_2_CO_3_ at 800–1000 °C, or Na_2_SO_4_ at 1200–1250 °C. In this study, the roasting stage was modified by using NaOH and avoiding the addition of sodium salts; hence, the operating temperature was significantly reduced to 400 °C as the melting point of NaOH was less than NaCl, Na_2_CO_3_, and Na_2_SO_4_. The amount of required energy for roasting was simply evaluated based on the heat for the increasing temperature of 1 kg of additives (Equation (15)) [26] and the heat for the transformation from the solid to the molten phase. The volumes of emitted gases were calculated with the assumption of the completed decomposition of sodium salts at the operating temperature.

Q = m × c × ΔT(15)

where Q: the heat kJ, m: the weight of the additive kg, c: specific heat kJ/kg.K, and ΔT: the change in temperature K.

The results in Table 4 show that roasting using NaOH requires the lowest amount of energy, and roasting using other additives consumes a greater amount of heat (1.5 to 3.3 times). This is mostly attributed to the value of the gradient temperature between the initial and operating temperature and the moderate heat capacity and heat of fusion of NaOH, although the mole of NaOH is the largest one. The decomposition of sodium salts induces large volumes of gas emissions: Cl_2_/191.5 L, CO_2_/210.5 L, and SO_2_/156.8 L, while using NaOH only releases H_2_O. Therefore, this method could minimize the adverse impacts on the environment caused by the emission of harmful gases and reduce the consumption of energy during roasting. Moreover, the combination of NaOH roasting and water leaching could selectively extract vanadium into the solution while titanium and iron remained in the leach residue based on the differences in the stabilities of their species in the alkaline media, which was necessary for the further purification stage. The recovery efficiency of vanadium was optimized to 98%, and the final products V_2_O_5_ and TiO_2_ could be simply obtained by using precipitation and hydrolysis processes with high purity ≥90%. It is worth investigating such a “greener process” at a larger scale and applying it to other available resources for the sustainable development of vanadium production.

## 4. Conclusions

The extraction of the value metals vanadium and titanium from domestic titanomagnetite with modification in roasting technique to reduce the consumption of energy and emission of harmful gases was investigated in the present study. NaOH roasting could effectively convert 90% of vanadium in the spinel phase to more soluble phases under the conditions: sample and NaOH weight ratio of 1:1, a temperature of 400 °C, a duration of 90 min. The roasted products were dissolved at 60 °C for 90 min and PD = 0.05 g/mL to selectively extract 98% of the vanadium from titanium and iron. The increase in temperature had a significant effect on the leaching of vanadium, and the kinetic study demonstrated that the leaching mechanism was controlled by the chemical reaction with high activation energy *E_a_*_(*leaching*)_ 69 kJ/mol. The vanadium in the leach liquor was further purified using NH_4_Cl in acidic media to produce the vanadium precipitate, which was calcinated at 550 °C to obtain vanadium pentoxide V_2_O_5_. Titanium in the residue after water leaching was dissolved in H_2_SO_4_ solution for the hydrolysis process, and the H_2_TiO_3_ was collected to prepare the titanium dioxide TiO_2_ by calcination at 800 °C. Both of the final products V_2_O_5_ and TiO_2_ presented high purities of 90% and 96%, respectively. The extraction process with modification in the roasting stage using NaOH is not only advantageous to reduce the roasting temperature but also to avoid the emission of harmful gases, which is suitable for the necessity of a cleaner approach for vanadium production.

## Figures and Tables

**Table 1:** Chemical composition of titanomagnetite concentrate.

Metals	Concentration (wt.%)
ΣFe	41.33
TiO_2_	23.28
V_2_O_5_	4.18
SiO_2_	6.25
Al_2_O_3_	4.35
CaO	2.54
MgO	4.37
Na_2_O	0.12
K_2_O	0.10
MnO	0.35
P_2_O_5_	0.18

**Table 2:** Fitting the leaching data to kinetic model equations [22].

Kinetic Model	Equation	Fitting Results	
		Average Value of Regression Coefficient R^2^	Active Energy *E_a_*_(*leaching*)_ (kJ/mol)
Film diffusion control ^a^	$\;x=\;k_{d}\;\times \;t$	0.97	54.4 kJ/mol
Ash diffusion control	1 − 3(1 − x)23+2(1 − x)= kd × t	0.91	99.2 kJ/mol
Chemical control	1 − (1 − x)13= kc × t	0.98	69.0 kJ/mol
Film diffusion control ^b^	1 − (1 − x)23= kd × t	0.98	61.1 kJ/mol

^a^ constant particle size; ^b^ shrinking particle; *x* is the leaching efficiency at certain time *t* (min); *k_d_* and *k_c_* are the apparent rate constants (min^−1^) for diffusion and chemical control.

**Table 3:** The chemical reactions during the recovery process.

Stage	Chemical Reactions	
NaOH roasting	4FeV_2_O_4_ + 8 NaOH + 5O_2_ → 8 NaVO_3_ + 2Fe_2_O_3_ + 4H_2_O	(2)
	4FeV_2_O_4_ + 16 NaOH + 5O_2_ → 4Na_4_V_2_O_7_ + 2Fe_2_O_3_ + 8H_2_O	(3)
	SiO_2_ + 2NaOH → Na_2_SiO_3_ +H_2_O	(4)
	TiO_2_ + 2NaOH → Na_2_TiO_3_ +H_2_O	(5)
Water leaching	NaVO_3_ + OH^−^→ HVO_4_^2−^+ Na^+^	(6)
	Na_4_V_2_O_7_ + H_2_O → 2 HVO_4_^2−^+ 4Na^+^	(7)
	Na_2_TiO_3_ + H_2_O → TiO_2_ + 2NaOH	(8)
Precipitation of vanadium	3H_2_V_10_O_28_^4−^ + 10NH_4_^+^ + 2H^+^ → 5(NH_4_)_2_V_6_O_16_↓ + 4H_2_O	(9)
Hydrolysis of titanium	FeTiO_3_ + 2H_2_SO_4_ → FeSO_4_ + TiOSO_4_ +2H_2_O	(10)
	TiO_2_ + H_2_SO_4_→ TiOSO_4_ + H_2_O	(11)
	TiOSO_4_ + 2H_2_O → H_2_TiO_3_↓ + H_2_SO_4_	(12)
Calcination	(NH_4_)_2_V_6_O_16_ → 3V_2_O_5_ + 2 NH_3_ + H_2_O	(13)
	H_2_TiO_3_ → TiO_2_ +H_2_O	(14)

**Table 4:** Comparison between modified roasting using NaOH and conventional roasting techniques using sodium salts.

Additives	Roasting Temperature (°C)	Specific Heat (J/mol.K)	Heat of Fusion (kJ/mol)	Q (kJ)	Heat for Melting (kJ)	Total	Gas/ Volume (L)
NaOH	400	59.5	8.4	557.8	210.0	767.8	None
NaCl	850	50.0	27.9	705.1	476.9	1182.0	Cl_2_/191.5
Na_2_CO_3_	1000	112.3	29.7	1032.9	280.2	1313.1	CO_2_/210.5
Na_2_SO_4_	1250	128.2	200.8	1105.9	1414.1	2520.0	SO_2_/156.8

## Data Availability

Not applicable.
